# Association Between Long-Term Proton Pump Inhibitor Use and the Risk of Osteoporotic Fractures

**DOI:** 10.3390/jcm15218398

**Published:** 2026-10-28

**Authors:** Aseel Oraby, Boris Khalfin, Sigal Fleisher-Berkovich, Shimon Ben-Shabat

**Affiliations:** Department of Clinical Biochemistry and Pharmacology, Faculty of Health Sciences, Ben-Gurion University of the Negev, P.O. Box 653, Beer-Sheva 8410501, Israel; orabya@post.bgu.ac.il (A.O.); boriskh83@gmail.com (B.K.); fleisher@bgu.ac.il (S.F.-B.)

**Keywords:** proton pump inhibitors, PPIs, osteoporosis, osteoporotic fractures, bone health, older adults, deprescribing, fracture risk

## Abstract

Proton pump inhibitors (PPIs) are among the most widely prescribed medications and remain a cornerstone in the treatment of acid-related gastrointestinal disorders. Their prolonged use, particularly among older adults, has raised concerns regarding a potential association with osteoporotic fractures. This review examines the current evidence linking long-term PPI therapy with fracture risk in older adults, explores the biological mechanisms proposed to explain this association, and discusses its clinical implications. Available epidemiological evidence, derived mainly from observational studies and meta-analyses, suggests a modest association between prolonged PPI use and increased risks of hip, vertebral, and overall osteoporotic fractures. Several mechanisms have been proposed, including impaired calcium absorption, altered mineral homeostasis, hypergastrinemia, micronutrient deficiencies, changes in bone remodeling and microarchitecture, and increased susceptibility to falls. However, evidence supporting these mechanisms is inconsistent, and causality remains uncertain. Although observational studies suggest an association between PPI use and fracture risk, a causal relationship has not been established. Overall, the relationship between long-term PPI use and fracture risk appears multifactorial and incompletely understood. Current evidence does not support routine discontinuation of PPIs when appropriately indicated, as their benefits generally outweigh potential skeletal risks. Instead, treatment should be individualized through regular reassessment of indication, use of the lowest effective dose, appropriate deprescribing when warranted, and assessment of fracture risk. Further prospective studies are needed to clarify causality, dose–response relationships, differences among individual PPIs, and the potential skeletal benefits of deprescribing.

## 1. Introduction

Proton pump inhibitors (PPIs) are widely used in the management of acid-related gastrointestinal disorders and have become a cornerstone of therapy for conditions such as gastroesophageal reflux disease, peptic ulcer disease, and Helicobacter pylori eradication regimens [1,2,3,4]. Their effectiveness and generally favorable short-term safety profile have also contributed to their frequent and, in some cases, prolonged use in clinical practice.

Due to their efficacy, PPIs are commonly used to treat gastroesophageal reflux disease (GERD), peptic ulcer disease, and *Helicobacter pylori* infection, and to prevent NSAID-induced gastrointestinal complications [1,2]. Their effectiveness and favorable short-term safety profile have led to extensive and often long-term use in clinical practice. Given their widespread use in clinical settings, it is important to examine patterns of long-term exposure to these medications [1,3,4].

Over the past decades, the use of PPIs has increased substantially, particularly among older adults [1,5,6]. A significant proportion of patients continue treatment beyond the recommended duration, often without reassessment of clinical need. In older populations, long-term exposure is especially common due to chronic gastrointestinal conditions and preventive prescribing practices, for example, gastroprotection in patients at increased risk of gastrointestinal bleeding who are receiving NSAIDs such as ibuprofen, naproxen, or antiplatelet therapy such as aspirin or clopidogrel [1,7].

This widespread use has raised concerns regarding potential long-term adverse effects. Among the various potential adverse outcomes, the impact of PPIs on bone health and fracture risk has become an increasingly important area of interest [3,5,6,8,9,10,11,12,13].

Osteoporotic fractures are a major cause of morbidity and mortality, particularly among older adults [14,15,16,17,18]. Hip fractures, vertebral fractures, and wrist fractures are the most common types and are associated with significant clinical consequences, including loss of independence, hospitalization, and increased mortality. The risk of fractures increases with age and is influenced by multiple factors, including bone mineral density, falls, comorbidities, and medication use [14,15,16,17]. Given the aging population, identifying modifiable risk factors for fractures is critical.

In this context, it is essential to explore whether commonly prescribed medications, such as PPIs, may contribute to fracture risk. A growing body of observational studies and meta-analyses suggests an association between long-term PPI use and an increased risk of osteoporotic fractures [5,12,13,19,20,21,22,23,24,25,26,27,28,29,30,31,32]. Reported estimates indicate a modest but consistent increase in fracture risk, with relative risks ranging from approximately 1.1 to 1.5, depending on fracture type and the population studied [20,22,23]. The association appears stronger for hip and vertebral fractures and may be influenced by factors such as duration of use, cumulative dose, and patient characteristics [13,19,21,25,26,27,33].

However, findings across studies are not entirely consistent, and some research has failed to demonstrate a significant association, highlighting the need for further clarification [21,33,34,35]. To better understand this potential relationship, it is important to consider the biological mechanisms that may underlie these findings.

Several biological mechanisms have been proposed to explain the association between PPI use and fracture risk. These include reduced calcium absorption due to decreased gastric acidity, leading to negative calcium balance; secondary hyperparathyroidism; and deficiencies in micronutrients such as vitamin B12 and magnesium, which may affect bone health and increase the risk of falls [6,10,36,37,38]. Additional mechanisms may involve direct effects on bone remodeling, alterations in bone microarchitecture, and indirect effects mediated through increased risk of falls [9,14,36,37].

Despite these hypotheses, the exact mechanisms remain incompletely understood. Given these uncertainties, further investigation is needed to clarify the clinical significance of the association between PPI use and fracture risk [5,10,12,13,34].

Therefore, given the widespread and often long-term use of PPIs, particularly among older adults, understanding their potential impact on bone health is of significant clinical importance. While many studies have examined the relationship between PPI use and fracture risk, inconsistencies in findings and limitations of observational data leave uncertainty regarding the strength and nature of this association [5,12,13,15,16,19,20,21,22,23,24,25,26,27,28,29,30,31,32,34,35,39].

This systematic review aims to summarize and critically evaluate the available literature on the association between long-term proton pump inhibitor use and osteoporotic fracture risk, with particular emphasis on the clinical, epidemiological, and mechanistic aspects of this relationship.

## 2. Proton Pump Inhibitors: Prescribing Patterns, Clinical Challenges, and Relevance in Older Adults

### 2.1. PPI Therapy and Its Growing Use in Older Adults

Proton pump inhibitors, such as omeprazole, pantoprazole, and esomeprazole, are among the most commonly prescribed medications worldwide and represent the most effective pharmacological therapy for suppressing gastric acid secretion [1,3,4,5]. By irreversibly inhibiting the H^+^/K^+^ ATPase enzyme in gastric parietal cells, PPIs block the final step of acid production and provide potent and prolonged acid suppression [1,2].

These medications are widely used to treat gastroesophageal reflux disease, peptic ulcer disease, *Helicobacter pylori* infection, and dyspepsia, and to prevent gastrointestinal complications associated with nonsteroidal anti-inflammatory drugs (NSAIDs) [1,2]. Because of their high efficacy and favorable short-term safety profile, PPIs have become a cornerstone of treatment for acid-related disorders [1,2].

Over recent decades, the use of PPIs has increased substantially, particularly among older adults [1,5,6]. Studies have shown that PPI use is highly prevalent among older individuals, with prevalence estimates ranging from approximately 15% to 40% among adults aged 65 years and older in community and hospitalized populations [1,6]. Chronic gastrointestinal symptoms, polypharmacy, and preventive prescribing practices contribute to the high prevalence of long-term therapy in this age group [1,6,7,40].

**Figure 1:**
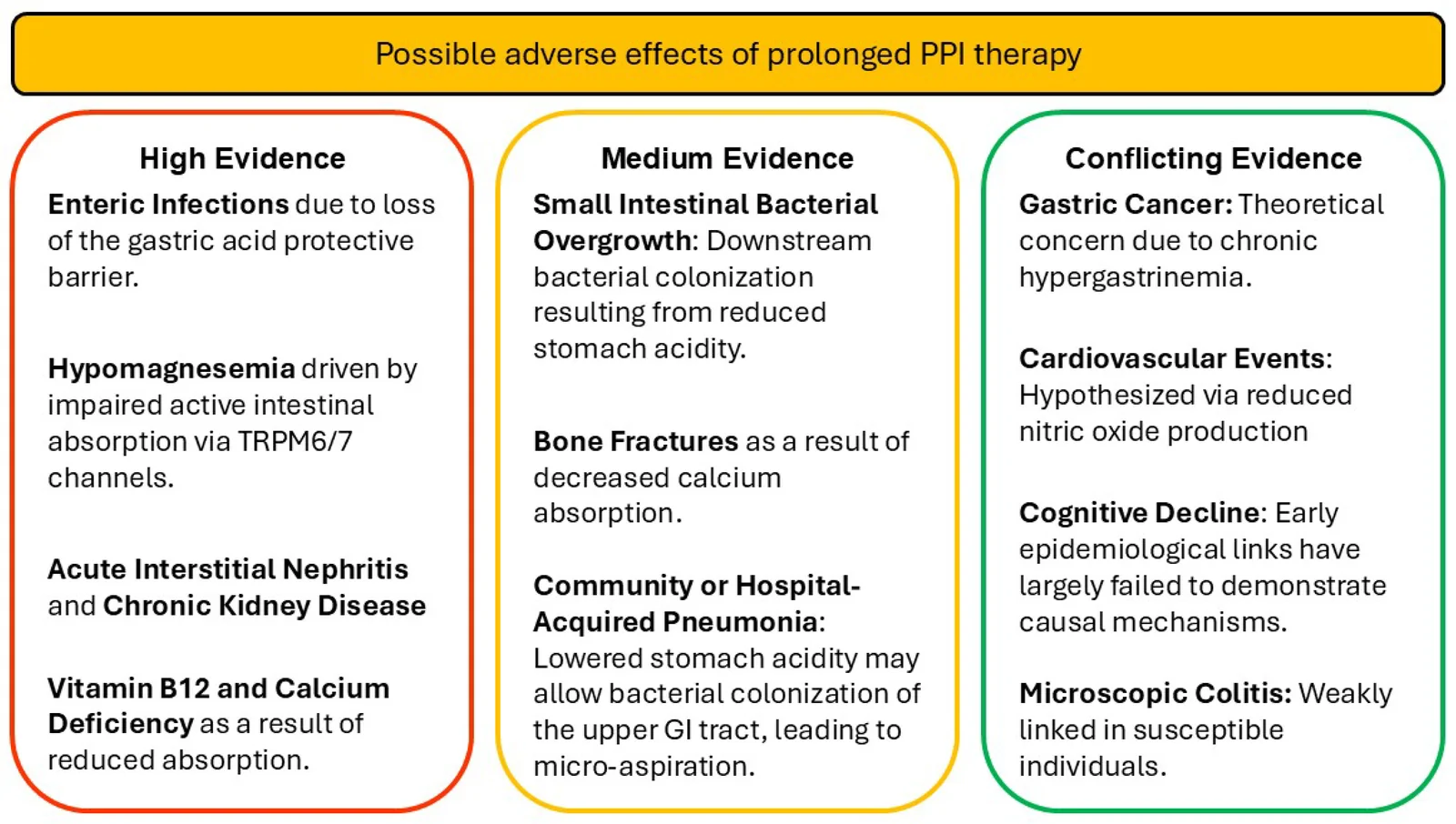
Potential adverse effects associated with long-term PPI therapy. The figure summarizes the adverse outcomes associated with prolonged PPI use and categorizes them according to the strength of the available evidence.

The growing prevalence of PPI therapy in aging populations has raised increasing concern regarding the long-term safety of these medications [3,4,5,6]. Although PPIs are generally considered safe when appropriately prescribed, long-term exposure has been associated with several potential adverse outcomes, including nutritional deficiencies, infections, renal complications, and possible effects on bone health [1,3,4,10,38]. The possible adverse effects are summarized in Figure 1.

Given the widespread use of PPIs among older individuals, it is important to evaluate whether prescribing practices consistently reflect evidence-based recommendations [1,2,7,40].

### 2.2. Inappropriate Prescribing and Long-Term PPIs Exposure

A substantial proportion of long-term PPI therapy may be unnecessary or continued beyond the appropriate clinical indication. Several studies suggest that many long-term PPI prescriptions may lack an appropriate evidence-based justification [1,7,40,41].

Inappropriate prescribing may include continuation of therapy beyond the recommended treatment duration, absence of regular reassessment, use without a confirmed clinical indication, or maintenance of high-dose therapy despite symptom resolution. In many cases, PPIs initiated during hospitalization or for short-term symptom relief are continued indefinitely without reevaluation of ongoing need. Although PPIs are prescription medications, chronic therapy may continue when prescriptions initiated for short-term indications are maintained without systematic reassessment of the original indication. Consequently, some patients may remain on PPI therapy for prolonged periods despite the absence of a clear ongoing clinical need [7,40,41].

This pattern appears particularly common among older adults. Chronic gastrointestinal symptoms, multiple comorbidities, and preventive use alongside NSAIDs or antiplatelet medications frequently contribute to extended therapy durations [1,3,6,7]. In addition, the perception that PPIs are relatively harmless medications may reduce the likelihood of treatment reassessment by both clinicians and patients [6,40,41].

However, concerns regarding the long-term safety of PPIs have increased in recent years. Observational studies have linked prolonged therapy to adverse outcomes such as vitamin B12 deficiency, hypomagnesemia, chronic kidney disease, infections, falls, and osteoporotic fractures [3,4,8,10,12,13,34,35,37,38,42]. Nevertheless, the strength and clinical significance of some of these associations remain controversial, and not all studies have demonstrated consistent findings [5,11,12,21,33,34,35].

These uncertainties highlight the importance of careful prescribing practices and regular evaluation of the ongoing need for therapy [1,7,40,41].

### 2.3. PPI Stewardship and Deprescribing Approaches

Growing awareness of the potential harms associated with long-term PPI therapy has led to increasing emphasis on PPI stewardship and deprescribing strategies [1,3,7,40].

PPI stewardship refers to the appropriate initiation, monitoring, dose adjustment, and discontinuation of therapy based on evidence-based clinical indications. International guidelines, including recommendations from NICE, AGA, and ACG, emphasize the importance of prescribing the lowest effective dose for the shortest necessary duration [1,2,7,40].

For patients with uncomplicated GERD or healed peptic ulcer disease, clinicians may consider dose reduction, intermittent treatment, on-demand therapy, or discontinuation after symptom control has been achieved. In contrast, long-term maintenance therapy remains appropriate for selected high-risk conditions, such as severe erosive esophagitis, Barrett’s esophagus, recurrent peptic ulcer disease, Zollinger–Ellison syndrome, or prevention of gastrointestinal bleeding in high-risk patients [1,2,7,40].

Despite these recommendations, deprescribing PPIs in clinical practice may be challenging. Many patients fear recurrence of symptoms after discontinuation, while clinicians may hesitate to stop treatment because of concerns regarding rebound acid hypersecretion or relapse of gastrointestinal disease [1,7,40].

Rebound acid hypersecretion refers to a temporary increase in gastric acid secretion following discontinuation of PPI therapy. This phenomenon may lead to recurrent gastrointestinal symptoms, making discontinuation more difficult for both patients and clinicians [1,7,40].

Furthermore, some patients remain on treatment for years without formal reassessment [7,40,41].

Several approaches have been proposed to reduce unnecessary exposure, including gradual dose tapering, transitioning to on-demand use, or switching to histamine-2 receptor antagonists (H2RAs). Lifestyle modifications, including dietary changes, weight reduction, and elevation of the head of the bed, may also support successful deprescribing efforts [2,7,40].

The importance of appropriate PPI stewardship becomes particularly significant in older adults, who are generally more vulnerable to medication-related adverse effects and complications [3,7].

### 2.4. Older Adults as a High-Risk Population for Adverse Outcomes

Older adults represent a particularly high-risk population in the context of chronic PPI therapy. Aging itself is associated with several physiological and clinical changes that increase susceptibility to osteoporosis and fractures, including reduced bone mineral density, sarcopenia, frailty, impaired balance, and increased fall risk [14,15,16,17,18].

In addition to age-related changes, older individuals frequently present with multiple comorbidities such as diabetes mellitus, chronic kidney disease, cardiovascular disease, and neurological disorders, all of which may independently contribute to impaired bone health and fracture risk. Polypharmacy is also highly prevalent in this population and may further increase the likelihood of falls and adverse drug interactions [3,14,15,16,17].

Alongside these age-related factors, long-term PPI therapy may introduce additional mechanisms that could negatively affect skeletal health. Proposed mechanisms include impaired calcium absorption due to reduced gastric acidity, hypomagnesemia, vitamin B12 deficiency, secondary hyperparathyroidism, and alterations in bone remodeling. Some studies have also suggested that PPIs may indirectly increase fracture risk by contributing to muscle weakness, gait disturbances, and falls [3,8,10,12,35,37,38].

Importantly, reduced bone mineral density (BMD) does not always directly correlate with fracture risk. Some studies have reported an increased risk of fractures despite minimal or inconsistent effects on BMD, suggesting that additional mechanisms, such as impaired bone quality or increased fall risk, may contribute to skeletal fragility among chronic PPI users [9,12,13,19,35,36,37].

However, the literature remains inconsistent regarding the magnitude and clinical relevance of these effects. While several observational studies and meta-analyses have reported an increased risk of osteoporotic fractures among long-term PPI users, other studies have shown weaker associations or failed to demonstrate significant effects on bone mineral density. These discrepancies may reflect differences in study populations, treatment duration, confounding factors, and methodological design [5,11,12,13,19,20,21,22,23,24,25,26,27,33,34,35].

Nevertheless, because older adults already have a high baseline risk of osteoporosis and fractures, even a modest medication-related increase in fracture risk may have important clinical implications [14,15,16,17,18].

Overall, the combination of widespread PPI use, frequent long-term exposure, and increased vulnerability among older adults highlights the importance of further examining the potential relationship between chronic PPI therapy and osteoporotic fracture risk [5,12,13,19,20,21,22,23,24,34,35].

## 3. Association Between PPI Use and Fracture Risk

### 3.1. Epidemiological Evidence Linking PPI Use to Fractures

Over the past two decades, increasing attention has been directed toward the possible association between long-term PPI therapy and osteoporotic fractures. Numerous observational studies, including cohort and case–control studies, as well as meta-analyses, have evaluated this relationship across different populations [5,12,13,19,20,21,22,23,24,25,26,27,28,29,30,31,32].

Several epidemiological studies have reported a modest but clinically relevant increase in fracture risk among long-term PPI users. Meta-analyses demonstrated increased risks of hip, vertebral, and overall osteoporotic fractures, with reported relative risks (RRs) generally ranging from approximately 1.1 to 1.5, depending on fracture type and study population [12,13,20,22,23]. The association appears to be more evident among older adults, who already have a higher baseline risk of osteoporosis and falls [14,15,16,17,18].

Some studies further suggest a possible dose–response relationship between long-term PPI therapy and fracture risk. Fracture incidence appears to increase with longer treatment duration, higher cumulative exposure, and high-dose therapy [13,19,21,22,23,25,26,27,33]. Several investigations reported that patients receiving PPI therapy for more than one year demonstrated higher fracture risk compared with short-term users [21,23,25,26,27]. These findings may support the possibility that prolonged gastric acid suppression progressively increases skeletal vulnerability [13,19,22,23].

However, despite the relatively consistent association reported in many studies, findings across the literature remain controversial [5,11,12,13,19,21,33,34,35].

Importantly, most available evidence is derived from observational studies. Therefore, these findings do not establish a direct causal relationship between PPI therapy and osteoporotic fractures [5,12,13,20,21,22,23].

In addition, several methodological limitations should be considered when interpreting these findings. Residual confounding may influence observed associations because long-term PPI users often have multiple comorbidities, polypharmacy, frailty, and baseline characteristics that independently increase fracture risk. Indication bias may also exist, as patients prescribed PPIs frequently differ clinically from non-users. Furthermore, reverse causation cannot be completely excluded in some studies [5,12,13,21,34,35].

Differences in study populations, treatment duration, adjustment for confounding variables, and methodological design may partly explain the variability in reported results [5,11,12,13,19,20,21,22,23,24,34,35].

Taken together, these limitations highlight the importance of examining fracture patterns and patient-related risk factors in greater detail. In addition, understanding the biological mechanisms that may link PPI therapy to impaired bone health may help explain these epidemiological observations [8,9,10,12,13,19,35,36,37,38].

### 3.2. Fracture Types Associated with Long-Term PPI Exposure

Several fracture types have been associated with long-term PPI exposure, although the strength of the association appears to differ across skeletal sites [5,12,13,19,20,21,22,23,24,25,26,27,28,29,30,31,32].

Hip fractures are among the most extensively studied outcomes because of their major clinical consequences in older adults, including disability, hospitalization, loss of independence, and increased mortality [14,15,16,17,18]. Several meta-analyses reported modestly increased risks of hip fractures among long-term PPI users [20,22,23], particularly in elderly populations (RR approximately 1.20–1.30) [20,22,23].

Vertebral fractures have also demonstrated relatively strong associations with long-term PPI therapy in several observational studies, with reported odds ratios (OR) ranging from approximately 1.50–1.56 [20,22,23]. These findings raise concerns regarding the potential effects of chronic acid suppression on bone quality and skeletal fragility [9,10,19,35,36,37].

Importantly, these epidemiological findings may partly reflect several biological mechanisms proposed in the literature, including impaired calcium absorption, micronutrient deficiencies, altered bone remodeling, and increased susceptibility to falls [8,9,10,14,35,36,37,38].

Beyond site-specific fractures, several studies have evaluated overall osteoporotic fracture risk and reported increased fracture incidence among long-term PPI users [13,20,22,23] (overall fracture risk approximately RR 1.16–1.20 [21]) [13,20,22,23]. However, substantial variability between studies remains evident. This variability likely reflects differences in patient characteristics, therapy duration, comorbidities, and concurrent medication use [5,12,13,19,20,21,22,23,24,25,26,27,33,34,35].

Because fracture risk appears to vary between patient populations, it is important to examine factors that may further modify skeletal vulnerability in older adults [14,15,16,17,18].

### 3.3. Magnitude of Risk in Older Adults

Older adults appear to represent the population most vulnerable to the potential skeletal effects of long-term PPI therapy. Several studies suggest that the association between long-term PPI use and fracture risk is more pronounced in elderly individuals compared with younger populations [5,13,19,20,21,22,23,24,25,26,27,28,29,30,31,32].

Meta-analyses focusing on older adults reported fracture risk estimates that were generally higher than those observed in the general population. One study reported fracture risk among older adults receiving PPI therapy of approximately OR 1.41, compared with estimates ranging from approximately RR 1.16–1.20 in broader adult populations. Even modest increases in fracture risk may have important clinical implications in elderly individuals because baseline fracture rates are already elevated in this age group [5,13,20,22,23].

Several investigations additionally suggested that fracture risk may progressively increase with longer treatment duration and cumulative exposure, further supporting the possibility of a dose–response relationship among long-term PPI users [13,19,21,22,23,25,26,27,33].

Hip and vertebral fractures in older adults are associated with substantial morbidity, reduced functional status, loss of independence, institutionalization, and increased mortality. Consequently, even relatively small medication-related increases in fracture risk may become clinically meaningful [14,15,16,17,18].

This increased vulnerability is likely reflected in the interaction between medication-related effects and age-related physiological changes. Older adults commonly experience reduced bone mineral density, sarcopenia, impaired balance, frailty, and increased susceptibility to falls. Therefore, additional skeletal or neuromuscular effects associated with long-term PPI exposure may further increase fracture risk [8,9,10,14,15,16,36,37,38].

However, not all studies demonstrated identical findings. Some studies reported weaker associations in specific subgroups of older adults, particularly among patients with advanced frailty or multiple competing risk factors. These inconsistencies further support the possibility that fracture risk associated with PPI therapy is multifactorial rather than exclusively medication-driven [11,12,21,33,34,35].

Accordingly, several demographic and clinical factors may influence the magnitude of fracture risk observed among long-term PPI users [14,16,21,24,34,35].

### 3.4. Factors Modifying Fracture Risk in Older Adults

The relationship between long-term PPI therapy and osteoporotic fractures appears to be influenced by multiple demographic, clinical, and treatment-related variables. Several studies suggest that fracture risk may be modified by treatment duration, cumulative exposure, sex, age, frailty, comorbidities, polypharmacy, and fall susceptibility [5,13,19,20,21,22,23,24,25,26,27,33,34,35].

These modifying factors may partly explain the heterogeneity observed across epidemiological studies. In addition, they highlight the complexity of distinguishing medication-related effects from baseline patient vulnerability in older populations [5,12,13,19,20,21,22,23,24,34,35].

The following sections summarize several major factors that may contribute to susceptibility to fracturing among long-term PPI users.

#### 3.4.1. Sex Differences

Several studies have demonstrated stronger associations between long-term PPI use and osteoporotic outcomes among women compared with men [8,9,19,26,43].

Women represent a substantial proportion of long-term PPI users and already have an increased baseline risk of osteoporosis because of age-related hormonal changes, particularly following menopause. Estrogen deficiency accelerates bone resorption and contributes to progressive reductions in bone mineral density [15,16,17,18].

Some investigations reported that women receiving long-term PPI therapy experienced higher rates of fractures, falls, and osteoporosis-related complications compared with men. In a recent pharmacovigilance analysis of osteoporosis-related adverse events reported among PPI users, women accounted for 61.9% of reports with known sex, compared with 17.9% for men. These data describe the distribution of spontaneous reports and should not be interpreted as evidence of a differential risk of osteoporosis or fractures between women and men [8]. Proposed explanations include longer treatment duration, greater cumulative exposure, and increased susceptibility to nutritional deficiencies affecting bone metabolism [8,10,37,38].

Nevertheless, findings regarding sex differences remain inconsistent, and some studies failed to demonstrate significant differences between males and females [11,19,21,33].

#### 3.4.2. Age, Frailty, and Comorbidities

Advanced age and frailty are major determinants of fracture risk in older populations. Aging is associated with progressive reductions in bone strength, muscle mass, mobility, and balance, all of which contribute to increased susceptibility to falls and fractures [14,15,16,17,18].

Older adults receiving long-term PPI therapy frequently present with multiple comorbidities, including chronic kidney disease, diabetes mellitus, cardiovascular disease, neurological disorders, and malignancy. Many of these conditions independently contribute to impaired bone health and increased fracture risk [3,14,15,16,34].

Frailty itself may also influence medication-related vulnerability. Patients with frailty often demonstrate poorer nutritional status, reduced physical reserve, and increased sensitivity to adverse drug effects. These factors may amplify the potential skeletal impact of long-term PPI exposure [3,14,34,35].

Long-term PPI exposure has been reported in observational studies, with median exposure durations of up to approximately 4.6 years in some cohorts. This estimate is derived from cohort-based observational data rather than from spontaneous pharmacovigilance reporting and should therefore be interpreted in the context of the underlying study design [8].

#### 3.4.3. Concurrent Medications and Polypharmacy

Polypharmacy is highly prevalent among older adults and may significantly influence fracture risk in patients receiving long-term PPI therapy [1,3,14,34].

Several medications commonly co-prescribed with PPIs have independent adverse effects on bone metabolism or fall risk. These include glucocorticoids, NSAIDs, opioids, antidepressants, benzodiazepines, and certain antihypertensive agents [1,14,16,17].

In particular, glucocorticoids are strongly associated with accelerated bone loss and osteoporotic fractures. Some evidence also suggests that interactions between PPIs and medications metabolized by CYP450 pathways may further increase skeletal risk [1,3,15,16].

Additionally, the cumulative burden of multiple medications may increase the risk of dizziness, impaired balance, muscle weakness, and falls, thereby indirectly increasing fracture risk [14,15,16,17,34].

#### 3.4.4. Falls as an Intermediate Pathway to Fracture

Falls are among the major contributors to osteoporotic fractures in older adults, particularly hip fractures [14,15,16,17,18].

Several mechanisms have been proposed through which long-term PPI therapy may indirectly increase fall risk. Long-term therapy has been associated with vitamin B12 deficiency (OR approximately 1.65) and hypomagnesemia (OR approximately 1.83). These abnormalities may contribute to muscle weakness, gait disturbances, neurological impairment, and impaired balance [10,14,34,35,37,38].

Importantly, this proposed pathway differs from the potential direct skeletal effects of PPIs on bone metabolism and bone quality. While some mechanisms suggest a direct influence on bone remodeling or mineral homeostasis, others suggest that PPIs may increase fracture risk indirectly by increasing susceptibility to falls. These indirect mechanisms may complement the direct skeletal mechanisms discussed in the following section, including impaired mineral absorption and altered bone remodeling [9,10,35,36,37,38].

However, these mechanisms remain incompletely understood and are not yet fully established. Current evidence mainly derives from observational studies and therefore cannot definitively determine the relative contribution of direct skeletal effects versus fall-related mechanisms [5,12,13,20,21,22,23,24,34,35].

Some studies reported increased rates of falls and fracture-related hospitalizations among older adults receiving long-term PPI therapy, particularly in postmenopausal women [14,26,43]. Nevertheless, further research is still needed to clarify these relationships [5,13,19,24,34,35].

Overall, the available epidemiological evidence suggests that the association between long-term PPI use and osteoporotic fractures is likely multifactorial and influenced by several interacting clinical variables [5,12,13,15,16,19,20,21,22,23,24,25,26,27,28,29,30,31,32,34,35,39].

From a clinical perspective, these findings emphasize the importance of careful risk–benefit assessment when prescribing long-term PPI therapy in older adults. Regular reassessment of treatment indication, duration, fall risk, and bone health may help reduce unnecessary exposure and improve patient safety in vulnerable elderly populations [1,2,7,14,16,17,18,40].

## 4. Proposed Mechanisms Linking PPI Use to Bone Fragility

The epidemiological findings presented in Section 3 suggest that long-term PPI therapy may be associated with an increased risk of osteoporotic fractures, particularly among older adults. However, observational associations alone cannot establish causality. Therefore, several biological mechanisms have been proposed to explain how prolonged gastric acid suppression may contribute to skeletal fragility and increased fracture susceptibility [5,12,13,19,20,21,22,23,24,25,26,27,34,35].

Importantly, these mechanisms may involve both direct effects on bone metabolism and indirect effects mediated through nutritional deficiencies, impaired neuromuscular function, and increased fall risk. As discussed in Section 3.4.4, some of the observed fracture risks may result not only from impaired bone strength itself, but also from increased susceptibility to falls among vulnerable older individuals [10,14,34,35,36,37,38].

**Figure 2:**
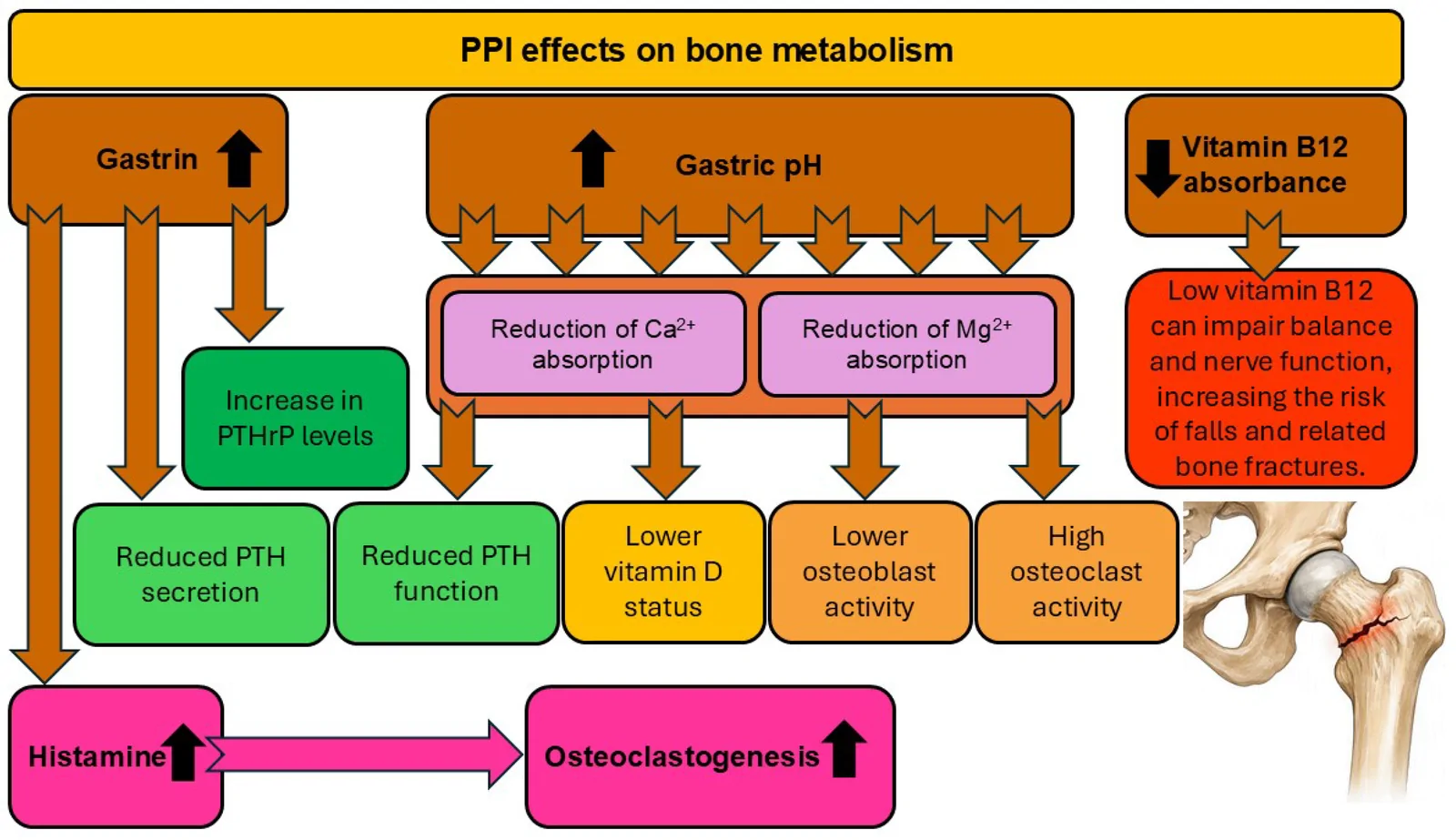
Potential mechanisms of proton pump inhibitor-induced fracture risk. The figure illustrates the principal biological pathways through which prolonged PPI therapy may contribute to skeletal fragility and fracture risk.

Although no single mechanism has been definitively established, the interaction between impaired mineral homeostasis, altered bone remodeling, micronutrient deficiencies, and neuromuscular dysfunction may collectively contribute to fracture risk in long-term PPI users [6,10,12,13,19,35,36,37,38]. Potential mechanisms of proton pump inhibitor-induced fracture risk are presented in Figure 2.

### 4.1. Altered Calcium Absorption and Mineral Homeostasis

One of the most commonly proposed mechanisms linking long-term PPI therapy to osteoporotic fractures involves impaired calcium absorption secondary to chronic gastric acid suppression [4,10,12,22,34,35].

PPIs increase gastric pH by inhibiting acid secretion in parietal cells. Gastric acidity normally facilitates the solubilization and intestinal absorption of calcium, particularly insoluble calcium salts such as calcium carbonate. Reduced gastric acidity may therefore impair calcium ionization and decrease intestinal calcium absorption [1,4,10,12,22,34].

Several studies have suggested that prolonged PPI therapy may contribute to negative calcium balance and secondary disturbances in mineral homeostasis. Chronic reductions in calcium absorption may stimulate compensatory increases in parathyroid hormone secretion, potentially leading to increased bone resorption over time [10,12,22,35,37].

Findings regarding calcium absorption and bone mineral density remain inconsistent. Some investigations demonstrated only modest effects on calcium metabolism, whereas others failed to identify significant reductions in bone mineral density despite increased fracture risk. These findings suggest that impaired calcium absorption alone may not fully explain the observed epidemiological associations [13,19,21,34,35,36].

Importantly, older adults may be particularly susceptible to these effects because aging itself is associated with reduced calcium absorption efficiency, vitamin D insufficiency, and declining bone mass [14,15,16,17,18].

The relationship between mineral imbalance and skeletal fragility is further supported by studies evaluating vitamin D, calcium, phosphate, and magnesium levels among older individuals receiving long-term PPI therapy [35,38].

In addition, altered calcium metabolism may contribute not only to impaired bone strength but also indirectly to muscle dysfunction and increased fall susceptibility, thereby further increasing fracture risk in elderly populations [14,35,37,38].

Nevertheless, despite being one of the most frequently cited mechanisms, evidence regarding the magnitude of impaired calcium absorption remains inconsistent. Several clinical studies failed to demonstrate substantial reductions in bone mineral density among long-term PPI users, suggesting that impaired calcium absorption alone may not fully explain the observed fracture risk [13,19,21,34,35,36].

**Figure 3:**
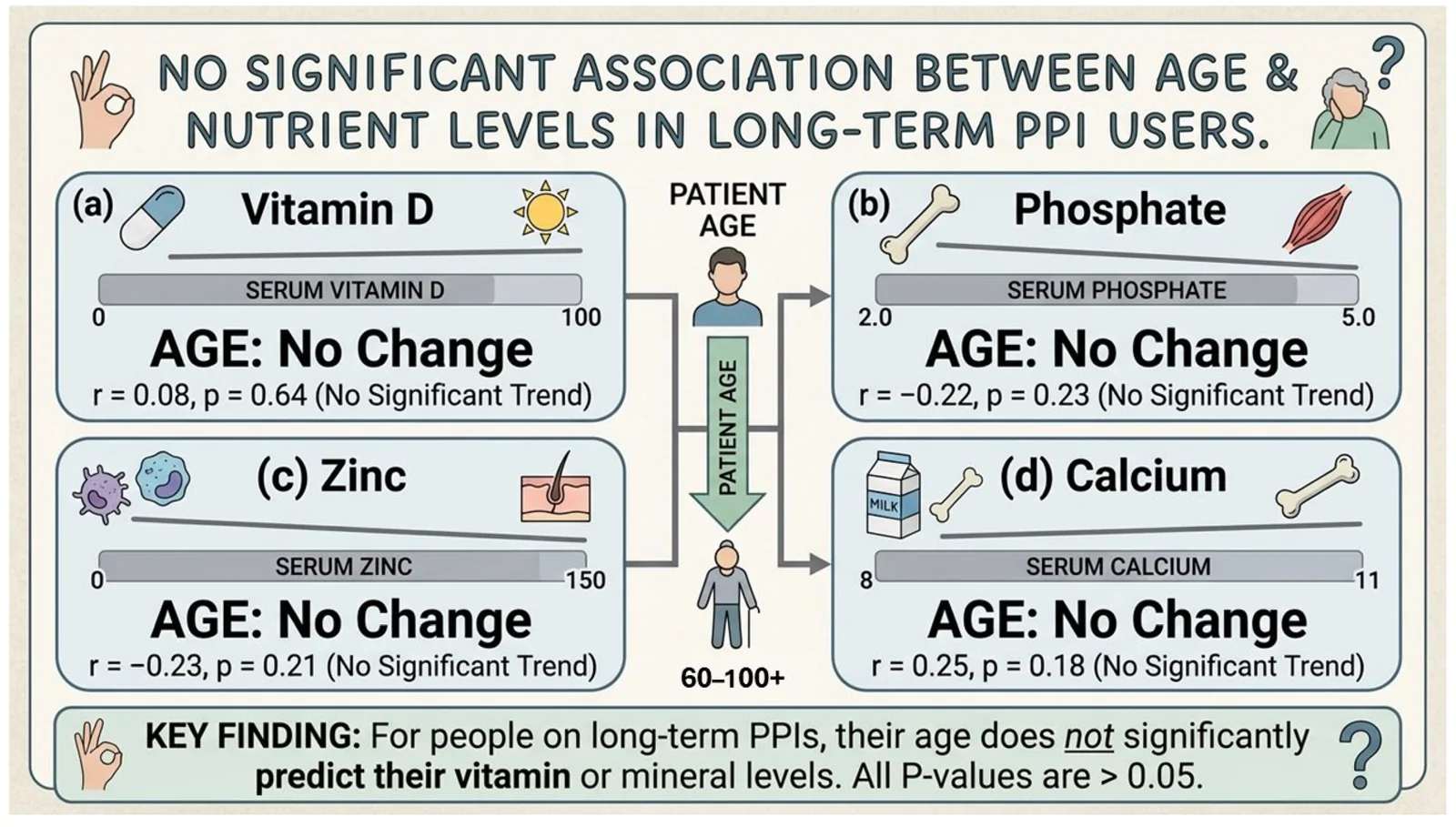
Schematic representation of age-related serum biomarker trends in long-term PPI users. Graphical summary illustrating the absence of statistically significant correlations between patient age (60–100+ years) and serum levels of vitamin D (**a**), phosphate (**b**), zinc (**c**), and calcium (**d**) among individuals on long-term PPI therapy [38]. Horizontal flat trend lines indicate that patient age does not significantly predict these biochemical parameters in this cohort.

Reduced calcium absorption may ultimately contribute to impaired bone mineralization and decreased skeletal strength. Over time, these changes may increase susceptibility to osteoporotic fractures, particularly in older adults with reduced bone reserves [10,12,13,22,35,37]. Schematic representation of age-related serum biomarker trends in long-term PPI users is depicted in Figure 3.

### 4.2. Hormonal Effects: Hypergastrinemia and Secondary Hyperparathyroidism

Beyond impaired mineral absorption, hormonal alterations associated with chronic gastric acid suppression may also influence bone metabolism and fracture susceptibility [4,10,12,22,37].

Long-term PPI therapy may induce hypergastrinemia due to feedback stimulation of gastrin secretion following sustained suppression of gastric acid production. Elevated gastrin levels have been hypothesized to indirectly influence bone metabolism by affecting parathyroid hormone regulation and osteoclast activity [6,10,12,22,37].

Some experimental studies suggested that hypergastrinemia may stimulate histamine release and increase osteoclastogenesis, potentially promoting bone resorption. Additional hypotheses propose that chronic PPI therapy may impair parathyroid hormone function and alter calcium homeostasis, thereby contributing to secondary hyperparathyroidism [10,12,22,35,37].

However, compared with other proposed mechanisms such as impaired calcium absorption and micronutrient deficiencies, the evidence supporting hypergastrinemia-induced skeletal effects remains relatively limited. Most available data originate from experimental studies, and direct clinical evidence demonstrating a significant contribution of hypergastrinemia to fracture risk in humans remains inconclusive. Therefore, this mechanism should currently be considered biologically plausible but still largely speculative [10,12,22,34,37].

Although these mechanisms remain incompletely understood, they may partly explain why some studies observed increased fracture risk even in the absence of substantial reductions in bone mineral density [12,13,19,34,35,36].

If these hormonal alterations lead to increased bone resorption over time, they may contribute to skeletal fragility and potentially increase fracture susceptibility. However, the clinical relevance of this pathway remains uncertain [10,12,22,34,37].

### 4.3. Micronutrient Deficiencies and Fall Risk (Vitamin B12, Magnesium)

In addition to hormonal alterations, chronic PPI use has been associated with deficiencies in several micronutrients involved in both bone metabolism and neuromuscular function [3,4,10,34,35,37,38].

Chronic gastric acid suppression may impair the absorption of vitamin B12, magnesium, calcium, and possibly other micronutrients involved in bone and neuromuscular function. Vitamin B12 deficiency has been associated with peripheral neuropathy, gait disturbances, muscle weakness, and impaired balance. Similarly, hypomagnesemia may contribute to muscle dysfunction, neurological symptoms, and impaired vitamin D metabolism [4,10,34,35,37,38].

Magnesium plays an important role in both bone metabolism and neuromuscular function. It is involved in vitamin D activation, calcium homeostasis, osteoblast activity, and maintenance of normal muscle function. Chronic hypomagnesemia may therefore adversely affect skeletal health through multiple pathways. In addition to impairing bone metabolism, magnesium deficiency has been associated with muscle weakness, fatigue, cramps, impaired balance, and an increased risk of falls. These effects may be particularly relevant in older adults, who already demonstrate age-related declines in muscle strength and physical function. Consequently, hypomagnesemia may contribute to fracture risk through both direct effects on bone health and indirect effects mediated by increased fall susceptibility [4,10,14,34,35,37,38].

These abnormalities may be particularly relevant in older adults, who already demonstrate increased vulnerability to frailty, sarcopenia, and falls [14,15,16,17,18].

Through their effects on muscle strength, balance, and neurological function, these deficiencies may increase the likelihood of falls. Since falls are a major precipitating factor for osteoporotic fractures in older adults, this pathway may indirectly contribute to increased fracture incidence [14,34,35,37,38].

The clinical significance of micronutrient deficiencies may also increase with prolonged treatment, as long-term exposure provides greater opportunity for their development and associated neuromuscular consequences [3,4,37,38].

As discussed in Section 3.4.4, observational studies reported increased odds of vitamin B12 deficiency (OR approximately 1.65) and hypomagnesemia (OR approximately 1.83) among long-term PPI users [8,37,38].

Similar findings have been reported in several systematic reviews and meta-analyses evaluating the long-term safety of PPI therapy. These studies consistently identified associations between prolonged PPIs exposure and deficiencies in vitamin B12, magnesium, and other micronutrients involved in bone and neuromuscular function, although the magnitude of risk varied across studies, and causality remains difficult to establish because most available evidence derives from observational research [3,4,22,34,37,38].

These findings support the hypothesis that nutritional and neuromuscular mechanisms may contribute in part to fracture risk [4,10,34,35,37,38].

Among the proposed mechanisms, micronutrient deficiencies and their effects on neuromuscular function are supported by relatively consistent observational evidence. They may provide a plausible explanation for the increased fall risk reported among long-term PPI users [14,34,35,37,38].

Importantly, this mechanism may help explain why some epidemiological studies observed increased fracture incidence despite inconsistent effects on bone mineral density itself [13,19,34,35,36].

However, most available evidence derives from observational studies, making it difficult to determine whether micronutrient deficiencies directly result from PPI therapy or reflect underlying comorbidities and frailty commonly observed among long-term PPI users [3,4,5,34,35,37,38].

### 4.4. Effects on Bone Density and Microarchitecture

Beyond micronutrient deficiencies and fall-related mechanisms, the relationship between long-term PPI therapy and bone mineral density remains controversial [9,12,13,19,34,35,36].

Several epidemiological studies reported increased fracture risk among chronic PPI users despite minimal or inconsistent reductions in BMD. Meta-analyses demonstrated fracture risk estimates ranging from approximately RR 1.16 to RR 1.50, depending on fracture type and duration of therapy [13,20,22,23].

These findings suggest that fracture susceptibility may involve additional alterations in bone quality and microarchitecture that are not fully captured by standard densitometry measurements [9,19,35,36].

Such alterations may weaken the bone’s structural integrity, increasing the likelihood of fractures even without substantial reductions in bone mineral density [9,19,35,36].

The observation that fracture risk may increase with prolonged treatment duration supports the hypothesis that long-term exposure could progressively influence skeletal integrity and bone quality [13,19,21,22,23,33].

Some experimental investigations suggested that chronic PPI exposure may influence trabecular bone structure, collagen integrity, and bone remodeling dynamics. Animal studies demonstrated possible reductions in bone mineralization and impaired skeletal microarchitecture following prolonged acid suppression [10,12,19,35].

However, clinical studies evaluating BMD in humans produced mixed findings. Some studies identified small reductions in BMD among long-term PPI users, whereas others failed to demonstrate statistically significant differences compared with non-users [9,13,19,21,34,35,36].

These inconsistencies may partly reflect differences in study design, treatment duration, patient age, baseline osteoporosis risk, nutritional status, concurrent medication exposure, and adjustment for potential confounding variables [5,11,12,13,19,21,34,35].

Importantly, fracture risk depends not only on bone mineral density but also on bone quality, microarchitecture, fall risk, and overall frailty. Therefore, PPI-related skeletal effects may involve a multifactorial process extending beyond simple reductions in BMD [9,14,15,16,17,19,35,36].

### 4.5. Direct Cellular Effects on Bone Remodeling

Beyond their potential effects on bone density and microarchitecture, PPIs may also exert direct effects on bone remodeling [10,12,19,35].

Bone remodeling depends on the balanced activity of osteoclasts and osteoblasts. Some laboratory studies proposed that PPIs may interfere with osteoclastic proton pumps, potentially altering bone resorption and remodeling dynamics [10,12,22,35].

Experimental models also suggested possible effects on osteoblast activity, osteoclastogenesis, and expression of inflammatory mediators involved in skeletal turnover. Furthermore, alterations in magnesium absorption and calcium signaling may indirectly influence osteoblastic function and bone formation [10,12,35,37,38].

If direct effects on bone remodeling occur in humans, their impact would likely develop gradually during prolonged exposure rather than during short-term therapy [10,12,22,35].

However, evidence supporting direct cellular effects on bone remodeling remains largely experimental, and its clinical relevance in humans has not been clearly established. Consequently, this mechanism currently has lower certainty than mechanisms involving mineral absorption or micronutrient deficiencies [10,12,22,34,35].

Furthermore, the available findings remain partially conflicting. Some studies proposed inhibitory effects on osteoclast activity that could theoretically protect bone, whereas others suggested impaired bone remodeling and reduced skeletal integrity [10,12,19,35].

If bone remodeling becomes impaired, the normal process of repairing microdamage within bone tissue may be disrupted. Over time, this could reduce skeletal resilience and increase susceptibility to fractures [9,10,12,19,35,36].

Most supporting evidence comes from laboratory and animal studies, and confirmation from large-scale human studies is currently lacking. This conclusion is consistent with several systematic reviews, which emphasize that many proposed mechanisms remain biologically plausible but have not been conclusively demonstrated in humans [10,12,22,34].

Therefore, although direct cellular effects remain biologically plausible, their precise contribution to fracture risk in humans remains uncertain [10,12,22,34,35].

The principal biological mechanisms proposed to explain the association between long-term PPI therapy and increased fracture risk are summarized in Table 1.

Overall, current evidence suggests that the association between long-term PPI therapy and osteoporotic fractures is likely multifactorial and results from the interaction of several biological pathways rather than a single mechanism [5,12,13,19,20,21,22,23,24,34,35].

Long-term gastric acid suppression may impair calcium and magnesium absorption, contributing to disturbances in mineral homeostasis and potentially reducing skeletal strength. At the same time, hormonal alterations such as hypergastrinemia and secondary changes in parathyroid hormone regulation may further influence bone metabolism. In parallel, deficiencies of vitamin B12 and magnesium may impair neuromuscular function, increase muscle weakness, reduce balance, and increase susceptibility to falls [10,12,14,22,34,35,37,38].

These mechanisms may interact with alterations in bone density, bone microarchitecture, and bone remodeling processes. Consequently, long-term PPI users may experience both increased skeletal fragility and increased fall risk. The combined effect of weaker bones and a greater likelihood of falling may ultimately contribute to the increased fracture risk observed in epidemiological studies, particularly among older adults with frailty, comorbidities, and polypharmacy [9,13,14,15,16,17,19,34,35,36].

Although the relative contribution of each mechanism remains uncertain, current evidence suggests that fracture risk is most likely the result of multiple interacting pathways acting simultaneously rather than a single isolated biological effect [5,10,12,13,19,20,21,22,23,24,34,35].

However, the strength of evidence supporting these mechanisms varies considerably. While impaired mineral absorption and micronutrient deficiencies are relatively well supported by clinical and observational data, other mechanisms, including hypergastrinemia-related skeletal effects and direct cellular effects on bone remodeling, remain less certain and require further investigation [10,12,22,34,35,37,38].

## 5. Clinical Implications and Practical Considerations

### 5.1. Risk–Benefit Assessment of PPI Therapy in Older Adults

PPIs remain among the most effective and widely prescribed medications for the management of acid-related gastrointestinal disorders, including gastroesophageal reflux disease, peptic ulcer disease, *Helicobacter pylori* eradication regimens, and prevention of gastrointestinal bleeding in high-risk patients. For many individuals, particularly those with established indications, the therapeutic benefits of PPIs clearly outweigh potential risks [1,2,7,40].

However, concerns have emerged regarding the long-term use of PPIs, especially among older adults. As discussed in previous sections, prolonged therapy has been associated with an increased risk of osteoporotic fractures, micronutrient deficiencies, hypomagnesemia, and other adverse outcomes. Older adults may be particularly vulnerable because aging itself is associated with declining bone mass, increased fall risk, frailty, polypharmacy, and multiple comorbidities [3,4,5,10,13,14,15,16,17,34,35,37,38].

Importantly, current evidence does not support routine discontinuation of PPIs in all older adults. Rather, treatment decisions should be individualized according to the balance between expected benefits and potential harms. Patients with clear indications for long-term therapy, such as severe destructive esophagitis, Barrett’s esophagus, chronic NSAID therapy with high gastrointestinal bleeding risk, or recurrent peptic ulcer disease, may derive substantial benefit from continued treatment despite potential adverse effects [1,2,7,40].

Quantifying the risk–benefit balance of long-term PPI therapy remains challenging because most available evidence on fracture risk comes from observational studies rather than randomized controlled trials. Consequently, reliable estimates of Number Needed to Treat (NNT) and Number Needed to Harm (NNH) are not consistently available. Nevertheless, current evidence suggests that for patients with established indications, including severe erosive esophagitis, Barrett’s esophagus, or prevention of gastrointestinal bleeding, the clinical benefits of PPIs generally outweigh the potential increase in fracture risk [1,2,5,7,13,20,21,22,23,40].

Importantly, older adults should not be considered a homogeneous population. Frail older individuals, nursing home residents, and patients with multiple comorbidities or polypharmacy may have a substantially higher baseline risk of falls and fractures than relatively healthy community-dwelling older adults. Therefore, treatment decisions should be individualized according to overall fracture risk, functional status, and expected clinical benefit [14,15,16,17,18,34].

Although fracture risk has received considerable attention, the absolute increase in fracture risk associated with PPI use appears relatively small compared with the substantial benefits derived from treatment in patients with established indications. Therefore, clinical decision-making should focus on individualized risk assessment rather than routine discontinuation based solely on concerns regarding skeletal health [1,2,7,13,20,21,22,23,40].

Conversely, prolonged therapy without an ongoing indication may expose patients to unnecessary risks without providing meaningful clinical benefit. Therefore, regular reassessment of treatment necessity is recommended, particularly among elderly patients receiving chronic PPI therapy [1,2,7,40,41].

### 5.2. Indications for Continuation vs. Discontinuation

Following the assessment of the potential benefits and risks of long-term PPI therapy, an important clinical question is whether treatment should be continued or discontinued.

Appropriate clinical decision-making requires periodic evaluation of whether ongoing PPI therapy remains necessary. Current evidence suggests that many patients continue PPIs treatment for prolonged periods despite the absence of a clear indication [1,2,6,7,40,41].

Continuation of long-term PPI therapy may be justified in patients with documented indications such as severe gastroesophageal reflux disease with recurrent symptoms, Barrett’s esophagus, chronic prevention of gastrointestinal bleeding in high-risk patients, Zollinger–Ellison syndrome, or persistent need for gastroprotection during treatment with NSAIDs or antithrombotic agents [1,2,7,40].

In contrast, discontinuation or dose reduction should be considered in patients whose original indication has resolved, particularly when treatment was initiated for uncomplicated dyspepsia, short-term ulcer healing, or transient reflux symptoms. Several studies have demonstrated that many patients can successfully reduce or discontinue PPI therapy without significant worsening of symptoms when appropriate deprescribing strategies are applied [1,7,40,41].

Because sudden discontinuation may lead to rebound acid hypersecretion and recurrence of symptoms, careful clinical assessment is required before treatment withdrawal. Shared decision-making between clinicians and patients is essential to ensure that potential benefits and risks are appropriately considered [1,7,40].

Current clinical guidelines support a personalized approach to long-term PPIs therapy. The American College of Gastroenterology (ACG) recommends maintenance therapy for patients with severe erosive esophagitis, Barrett’s esophagus, and other conditions requiring chronic acid suppression. Similarly, the American Gastroenterological Association (AGA) emphasizes regular reassessment of treatment indications while recognizing that many patients derive substantial benefit from continued therapy. National Institute for Health and Care Excellence (NICE) guidelines also recommend periodic review of the necessity of treatment and the use of the lowest effective dose whenever possible [1,2,7,40].

In addition, several studies have suggested that fracture risk may increase with both treatment duration and cumulative exposure. The highest risk estimates have generally been reported among individuals receiving prolonged therapy for more than one to two years, supporting the importance of periodic reassessment of treatment necessity [13,19,20,21,22,23,25,26,27,33].

### 5.3. Deprescribing Strategies in Clinical Practice

When discontinuation or dose reduction is considered appropriate, evidence-based deprescribing strategies may help minimize unnecessary medication exposure while maintaining adequate symptom control [1,2,7,40,41].

Deprescribing is the planned process of reducing or discontinuing medications that may no longer provide sufficient benefit or contribute to adverse outcomes. In recent years, deprescribing has become an important component of medication optimization in older adults [7,40,41].

Several approaches have been proposed for PPI deprescribing. One strategy involves gradual dose reduction, whereby the PPI dose is progressively decreased over time before complete discontinuation. Another approach consists of switching from continuous daily therapy to intermittent or on-demand use. In selected patients, alternative therapies such as histamine-2 receptor antagonists may also be considered [1,2,7,40].

Evidence from deprescribing studies suggests that many patients can successfully discontinue inappropriate PPI therapy while maintaining acceptable symptom control. Educational interventions, patient counseling, decision-support tools, and physician training have all been identified as factors that improve the success of deprescribing [1,7,40,41].

Reported rates of successful PPI discontinuation without significant symptom deterioration have ranged from approximately 14% to 64% across different studies, depending on patient characteristics, underlying indications, and the deprescribing strategy employed. Patients with uncomplicated gastroesophageal reflux disease generally demonstrate higher success rates than those with severe esophageal disease or persistent symptoms [1,7,40,41].

Although deprescribing may reduce unnecessary medication exposure, there is currently insufficient evidence to conclude that PPI discontinuation directly reduces fracture risk or improves bone mineral density. Most available studies evaluated symptom control and medication use rather than long-term skeletal outcomes [1,7,40,41].

Nevertheless, clinicians should remain aware of rebound acid hypersecretion, which may occur following PPI withdrawal and can temporarily mimic recurrence of the underlying disease. Patients should therefore receive appropriate education regarding expected symptoms and strategies for symptom management during the deprescribing process [1,7,40].

Overall, deprescribing should be individualized according to patient characteristics, treatment indication, symptom severity, and risk profile. The primary objective is not necessarily complete discontinuation, but rather achievement of the lowest effective dose required to maintain symptom control [1,2,7,40,41].

### 5.4. Monitoring and Prevention of Fracture Risk

Beyond decisions regarding continuation or deprescribing, clinicians should also consider strategies aimed at minimizing skeletal complications among patients who require long-term PPI therapy [1,2,7,40].

Given the growing evidence linking prolonged PPI therapy with increased fracture risk, appropriate monitoring and preventive measures may be particularly important in patients receiving long-term treatment [5,12,13,19,20,21,22,23,24,34,35].

Assessment of fracture risk should include multiple factors, such as age, sex, history of fractures, osteoporosis risk factors, comorbidities, fall risk, nutritional status, and duration of PPI exposure. Because fracture risk appears to result from multiple interacting mechanisms rather than a single biological pathway, a comprehensive clinical evaluation is required [14,15,16,17,18].

Particular attention should be paid to potential micronutrient deficiencies. Monitoring serum magnesium and vitamin B12 levels may be considered in selected patients receiving prolonged therapy, particularly in older adults and those with additional risk factors for deficiency. Adequate calcium and vitamin D intake should also be encouraged, in line with current osteoporosis prevention guidelines [15,16,17,37,38].

For patients at elevated risk of osteoporosis, bone mineral density assessment may be appropriate. Although studies evaluating the relationship between PPI therapy and bone mineral density have produced inconsistent findings, recent evidence suggests that long-term PPI use may affect aspects of bone quality and microarchitecture that are not fully captured by conventional densitometry alone [9,13,19,34,35,36].

The relationship between PPI dose, treatment duration, and fracture risk should also be considered during clinical assessment. Several observational studies have reported higher fracture risk estimates among patients receiving prolonged therapy and, in some cases, higher cumulative doses. However, the available evidence remains inconsistent, and the precise dose–response relationship has not been fully established [13,19,20,21,22,23,25,26,27,33].

Similarly, evidence comparing different PPIs remains limited. Although some studies suggested variations in fracture risk estimates among individual PPIs, current evidence is insufficient to conclude that one specific PPI agent is consistently safer than another with respect to bone health. Therefore, treatment selection should continue to be based primarily on clinical indication and patient-specific factors rather than presumed differences in skeletal safety [3,5,12,13,34].

Fall prevention strategies represent another important component of fracture risk reduction. Maintaining physical activity, balance training, optimizing vision, managing polypharmacy, and correcting nutritional deficiencies may all help reduce fracture risk among older adults [14,15,16,17,18].

Ultimately, prevention strategies should focus not only on preserving bone health but also on minimizing fall risk and maintaining overall functional status [14,15,16,17,18].

It should also be acknowledged that most available data linking PPI therapy to fracture risk originates from observational studies. Such studies are susceptible to residual confounding, indication bias, and differences in baseline health status between PPI users and non-users. Furthermore, long-term randomized controlled trials specifically designed to evaluate fracture outcomes are lacking. Consequently, although the observed associations are biologically plausible and supported by multiple studies, a direct causal relationship has not been conclusively established [5,12,13,19,20,21,22,23,24,25,26,27,34,35].

## 6. Conclusions and Future Directions

### 6.1. Summary of Key Findings

PPIs remain among the most effective pharmacological therapies for managing acid-related gastrointestinal disorders and continue to play a central role in the treatment of gastroesophageal reflux disease, peptic ulcer disease, *Helicobacter pylori* eradication, and the prevention of gastrointestinal bleeding in high-risk patients. As discussed in Section 1, their widespread use has substantially improved patient outcomes and quality of life, particularly among older adults [1,2,7,40].

The epidemiological evidence reviewed in Section 3 consistently suggests an association between prolonged PPI therapy and an increased risk of osteoporotic fractures, although the magnitude of this association varies across studies, and a definitive causal relationship has not been established [5,12,13,19,20,21,22,23,24,25,26,27,28,29,30,31,32,34,35]. The biological mechanisms presented in Section 4 indicate that this association is likely multifactorial. Impaired calcium and magnesium absorption, vitamin B12 deficiency, hormonal alterations, changes in bone remodeling, deterioration of bone quality, and increased susceptibility to falls may all contribute to fracture risk [10,12,14,19,22,34,35,36,37,38]. However, the strength of evidence differs among these mechanisms. While impaired mineral absorption and micronutrient deficiencies are supported by relatively consistent clinical and observational evidence, mechanisms involving hypergastrinemia and direct cellular effects on bone remodeling remain less certain and require further investigation [10,12,22,34,35,37,38]. 

Randomized evidence provides an important counterpoint to observational literature. In the COMPASS trial, Moayyedi et al. randomized 17,598 participants to pantoprazole 40 mg once daily or placebo and followed them for a median of approximately three years. Pantoprazole use was not associated with an increased risk of fractures compared with placebo, providing no evidence of a detrimental effect on fracture risk during the period studied. These findings are particularly relevant because randomization reduces the potential for confounding in observational studies. However, the relatively limited follow-up duration and the characteristics of the study population should be considered when extrapolating these findings to individuals receiving PPIs for substantially longer periods or with established long-term indications. Thus, while the COMPASS trial does not support an increased fracture risk with pantoprazole over approximately three years, it does not fully resolve the question of potential effects associated with longer-term exposure [44].

Taken together, these findings provide the basis for important clinical considerations regarding the appropriate use of long-term PPI therapy in older adults [1,2,7,14,40].

### 6.2. Clinical Implications for Practice

The clinical implications discussed in Section 5 emphasize that the potential skeletal risks associated with long-term PPI therapy should always be interpreted in the context of its well-established therapeutic benefits. Current clinical guidelines do not recommend routine discontinuation of PPIs solely because of concerns regarding fracture risk. Instead, treatment decisions should be individualized according to each patient’s clinical indication, expected therapeutic benefit, fracture risk, comorbidities, functional status, and overall risk profile.

Regular reassessment of the necessity of treatment, prescribing the lowest effective dose, implementing evidence-based deprescribing strategies when appropriate, optimizing calcium and vitamin D intake, correcting micronutrient deficiencies, and implementing fall-prevention strategies represent important components of the clinical management of older adults requiring prolonged PPI therapy [1,2,7,14,15,16,17,37,38,40,41]. Such an individualized approach may help maximize therapeutic benefit while minimizing potential adverse outcomes [1,2,7,14,40].

### 6.3. Directions for Future Research

Despite these important clinical implications, several important uncertainties remain regarding the long-term skeletal effects of PPI therapy. Most studies evaluating the association between long-term PPI therapy and fracture risk are observational and therefore remain susceptible to residual confounding, indication bias, and differences in baseline patient characteristics. Consequently, although the observed association is biologically plausible and supported by multiple epidemiological studies, a direct causal relationship has not yet been conclusively established [5,12,13,19,20,21,22,23,24,25,26,27,28,29,30,31,32,34,35].

Future research should focus on well-designed prospective studies to clarify the dose–response relationship between PPI therapy and fracture risk, determine whether individual PPIs differ in their effects on bone health, evaluate whether deprescribing reduces fracture risk or improves skeletal outcomes, and identify patient populations that may benefit most from individualized preventive strategies. Additional research is also needed to better define the relative contribution of the different biological mechanisms proposed to explain PPIs-associated skeletal fragility [13,19,24,31,32,34,35,41].

Overall, the evidence presented throughout this review suggests that long-term PPI therapy may be associated with an increased risk of osteoporotic fractures, particularly among older adults [5,12,13,19,20,21,22,23,24,25,26,27,28,29,30,31,32,34,35]. Nevertheless, PPIs remain highly effective medications whose clinical benefits continue to outweigh their potential risks when prescribed for appropriate indications [1,2,7,40]. Therefore, future clinical practice should emphasize individualized risk-benefit assessment, regular review of the necessity of treatment, and implementation of evidence-based preventive strategies to optimize patient outcomes while minimizing potential skeletal complications [1,2,7,14,15,16,17,40,41].

## Figures and Tables

**Table 1:** Summary of the Proposed Biological Mechanisms Linking Long-Term Proton Pump Inhibitor Therapy to Increased Fracture Risk. Data summarized and adapted from references [10,12,13,14,19,22,34,35,36,37,38].

Mechanism	Proposed Effect	Potential Impact on Fracture Risk	Level of Evidence
Impaired calcium absorption	Reduced calcium bioavailability and mineral homeostasis	Reduced bone mineralization and increased skeletal fragility	Moderate to Strong
Hypomagnesemia	Impaired bone metabolism and neuromuscular function	Increased fall risk and fracture susceptibility	High
Vitamin B12 deficiency	Neurological impairment, gait disturbances, and balance problems	Increased susceptibility to falls	High
Hypergastrinemia	Possible stimulation of bone resorption pathways	Potential contribution to skeletal fragility	Limited
Altered bone microarchitecture	Changes in bone quality independent of BMD	Increased fracture susceptibility	Moderate
Direct cellular effects on bone remodeling	Altered osteoblast and osteoclast activity	Possible impairment of bone remodeling	Limited
Increased fall risk	Greater likelihood of trauma-related fractures	Indirect increase in fracture incidence	Moderate to Strong

## Data Availability

No new data were created or analyzed in this study. Data sharing is not applicable to this article.

## Abbreviations

The following abbreviations are used in this manuscript:

ACG	American College of Gastroenterology
AGA	American Gastroenterological Association
BMD	Bone mineral density
GERD	Gastroesophageal reflux disease
NICE	National Institute for Health and Care Excellence
NNH	Number Needed to Harm
NNT	Number Needed to Treat
NSAID	Nonsteroidal anti-inflammatory drug
OR	Odds Ratio
PPI	Proton pump inhibitors
PTH	Parathyroid hormone
RR	Relative Risk
